# A Type I Collagen-Targeted MR Imaging Probe for Staging Fibrosis in Crohn’s Disease

**DOI:** 10.3389/fmolb.2021.762355

**Published:** 2021-11-11

**Authors:** Zhoulei Li, Baolan Lu, Jinjiang Lin, Shaofu He, Li Huang, Yangdi Wang, Jixin Meng, Ziping Li, Shi-Ting Feng, Shaochun Lin, Ren Mao, Xue-Hua Li

**Affiliations:** ^1^ Department of Radiology, The First Affiliated Hospital, Sun Yat-Sen University, Guangzhou, China; ^2^ Department of Gastroenterology, The First Affiliated Hospital, Sun Yat-Sen University, Guangzhou, China

**Keywords:** fibrosis, ColI-targeted theranostic probe, Crohn’s disease, molecular imaging, type I collagen

## Abstract

Fibrostenosis is a serious complication of Crohn’s disease (CD), affecting approximately one-half of all patients. Surgical resection is the typical clinical end due to ineffective antifibrotic therapy mainly through anti-inflammatory treatment and fibrosis can be reverted only at early stages. Mover, human fibrotic disorders is known to be associated with aging process. Thus, accurate monitoring of the progression of fibrosis is crucial for CD management as well as can be benefit to aging related fibrosis. The excessive deposition of type I collagen (ColI) is the core point in major complications of fibrosis, including that in patients with CD and aging related fibrosis. Therefore, a MR imaging probe (EP-3533) targeted ColI was employed to stage bowel fibrosis in CD using a rat model and to compare its efficiency with the common MR imaging contrast medium gadopentetatedimeglumine (Gd-DTPA). The bowel fibrotic rat model was established with different degrees of bowel fibrosis, were scanned using a 3.0-T MRI scanner with a specialized animal coil. MRI sequence including *T*_*1*_ mapping and T1-weighed imaging were performed before and after injecting the MRI probe (EP-3533 or Gd-DTPA). The *T*_*1*_ relaxation time (*T*_*1*_ value) and change in the contrast-to-noise ratio (ΔCNR) were measured to evaluate bowel fibrosis. Masson’s trichrome staining was performed to determine the severity of fibrosis. EP-3533 offered a better longitudinal relaxivity (r_1_) with 67.537 L/mmol·s, which was approximately 13 times that of Gd-DTPA. The *T*_*1*_ value on bowel segments was reduced in the images from EP-3533 compared to that from Gd-DTPA (F = 16.478; *p* < 0.001). Additionally, a better correlation between ΔCNR calculated from EP-3533 imaging and bowel fibrosis (AUC = 0.846) was determined 10 min after enhanced media administration than with Gd-DTPA (AUC = 0.532). The 10th-minute ΔCNR performed using the ColI probe showed the best correlation with the severity of bowel fibrosis (*r* = 0.538; *p* = 0.021). Our results demonstrates that targeted MRI probe (EP-3533) supplies a better enhanced effect compared to Gd-DTPA and could be a promising method to evaluate the progression and monitor the therapeutic response of bowel fibrosis.

## Introduction

Fibrostenosis is a serious complication of Crohn’s disease (CD), affecting approximately one-half of all patients (Rieder et al., 2016; Bettenworth et al., 2019). The main characteristic of CD fibrosis is the excessive deposition of extracellular matrix (ECM), such as collagen, within the intestinal wall. The current anti-inflammatory treatment can not prevent or reverse intestinal fibrosis (Rieder et al., 2018). Surgical resection is the typical clinical end of CD fibrostenosis. Mover, human fibrotic disorders is known to be associated with aging process (Newgard and Sharpless, 2013; Ferrucci et al., 2020; Rehan et al., 2021). Accurate monitoring the progression of intestinal fibrosis is crucial for CD management (Rieder et al., 2016), and could also benefit to management of aging related Fibrosis.

The major complication of fibrosis is normally caused by excessive deposition of ColI from the ECM (Karsdal et al., 2015; Makarev et al., 2016). The disease of CD is progressive and recurrent. However, there is no therapeutic approach to directly and specifically target ColI synthesis. Fibrosis can be reverted only at the early stage when collagen fibres are still in an unstable crosslinked state (Issa et al., 2003; Friedman and Bansal, 2006). The goal of optimal antifibrotic therapy is to inhibit ColI production only in fibrotic lesions and spare constitutive ColI synthesis. Because a therapeutic approach against fibrosis must be applied for prolonged periods of time with minimal side effects, it should specifically target excessive ColI synthesis and be affordable. The ColI is one of the most stable proteins in the human body, with a half-life of 4–12 months. Its fractional synthesis rate (defined as % synthesis per day) is very low in healthy organs, in contrast to synthesis in fibrosis, where ColI production can be increased several hundred-fold (Stefanovic et al., 1997; Lindquist et al., 2000). Additionally, ColI was also demonstrated to play an important role in aging related fibrosis (Randles et al., 2021; Rawlings et al., 2021). Therefore, ColI may be a target for the diagnose and treatment of tissue fibrosis, including that in patients with CD as well as aging related fibrosis.

To evaluate the therapeutic efficacy of drugs against fibrosis in CD, several specific challenges should be solved. First, compared with inflammation, which can be successfully assessed by endoscopic examination and is closely correlated with cross-sectional imaging (Stidham and Cross, 2016), routine endoscopic examination is insufficient to diagnose or analyze fibrotic strictures and the severity of fibrosis. Second, the diagnosis of fibrotic strictures is a key characteristic to select patients for inclusion in studies of antifibrotic drugs. Third, accurate clinical endpoints for studies of CD treatment remain to be identified and validated (Bettenworth et al., 2019; Panés et al., 2011). Non-invasive imaging techniques such as CT, MRI and ultrasound (US) are likely to provide the most tractable solution to these challenges (Panés et al., 2011). However, current techniques are more suitable for diagnosis of inflammation than fibrosis (Bettenworth et al., 2019). Therefore, an accurate imaging technique for fibrosis analysis is desired. Recently, several studies have also shown that the use of ColI-targeted imaging probe in MR imaging can benefit the fibrosis diagnosis in Duchenne muscular dystrophy (Murphy et al., 2021), nonalcoholic steatohepatitis (Zhou et al., 2020), diffuse cardiac fibrosis (Ezeani et al., 2021) and pancreatic cancer (Polasek et al., 2017). In this study, we generated an CD fibrosis rat model to evaluate the strategy of targeting ColI for staging fibrosis in CD and to compare its efficiency with Gd-DTPA, which is a common MR imaging contrast medium.

We demonstrated that the ColI-targeted MR imaging probe is more effective in the detection of progression of fibrosis than the Gd-DTPA. This probe could also be suitable for monitoring the therapeutic response of tissue fibrosis.

## Materials and Methods

### Animal Model

To decrease the influence of confounding factors and mimic the progression of bowel fibrosis, we generated a rat CD fibrosis model. This study was approved by the institutional ethics review board of our university (approval number: [2018]237). All experiments were performed in accordance with the ethics regulations of animal research. The Sprague-Dawley rats were administered 150 mg/kg of 2,4,6-trinitrobenzene sulfonic acid (TNBS) (1 M; 293.17 mg/ml; Sigma Aldrich, St Louis, Missouri, United States) once weekly at the second (*n* = 5), third (*n* = 12), or fourth (*n* = 5) week after initiation of the experiment to induce bowel fibrosis with different degree. Thirteen rats were imaged by administering EP-3533, and the other nine rats were imaged by treatment with Gd-DTPA as a control for enhanced imaging.

### Synthesis of Collagen-Targeted Molecular Probe

EP-3533 comprises a 16-amino-acid peptide with a 10-amino-acid disulphide bridge cyclic core conjugated to three gadolinium (Gd) moieties and was synthesized as previously reported (Caravan et al., 2007). Chemical identification of purified EP-3533 was performed using mass spectrometry and elemental analysis. The longitudinal relaxivity (r_1_) was reported to be measured at 3.0-T field strengths in pH 7.4 phosphate-buffered saline (PBS) or in human plasma (Caravan et al. (2007)), since we determined r_1_ of EP-3533 at 3.0-T field strengths in aqueous solution. The analysis of peptide affinity (Kd = 1.8 μM) and biodistribution in rat for ColI was performed as described by Caravan et al. (2007).

### MR Imaging

Animals were imaged approximately 1 week after the last enema to avoid the influence of acute inflammation. They were fasted for 24 h before MR scanning. The rats were anaesthetized using an intraperitoneal injection of 2% pentobarbital sodium (30 mg/kg) and intramuscular injection of raceanisodamine hydrochloride (0.1 mg) (Minsheng Pharma, Hangzhou, China) to minimize intestinal peristalsis. The tail vein was cannulated for intravenousdelivery of the contrast agent while the animal was positioned in the scanner. MR examination was performed using a 3.0-T MR scanner (Magneton Verio; Siemens, Erlangen, Germany) with a 4-channel animal coil (Chenguang Medical Technologies, Shanghai, China). Each animal was placed within the solenoid coil in the supine position.

Routine MR images were performed using a 2 mm slice thickness, no inter-slice gap in the following sequences: sagittal T2-weighted (TE = 99 ms; TR = 4,000 ms; field of view (FOV) = 70 mm × 100 mm; matrix = 180 × 256), coronal T2-weighted (TE = 99 ms; TR = 4,000 ms; FOV = 70 mm × 100 mm; matrix = 180 × 256) followed by axial T1-weighed (TE = 15 ms; TR = 700 ms; FOV = 49 mm × 70 mm; matrix = 180 × 256) and T2-weighted (TE = 99 ms, TR = 3,200 ms, FOV = 49 mm × 70 mm, matrix = 180 × 256).

A three-dimensional-volume interpolated body examination (3D-vibe) T1-weighted dynamic sequence with high spatial resolution was used to analyse the EP-3533 in MR imaging. 3D-T_1_WI were obtained at ten-minute intervals, starting at the time point of EP-3533 (30 μmol/kg) or Gd-DTPA (30 μmol/kg) administration. Image acquisition parameters comprised the following parameters: TE = 3.6 ms; TR = 9.2 ms; field of view (FOV) = 52 mm × 70 mm; matrix = 192 × 256; slice thickness = 2.0 mm. To generate R1 relaxation rate maps, a series of dual flip-angle (2° and 12°) three-dimensional (3D) gradient echo sequences were also acquired before and 10, 20, 30, 40, 50 and 60 min following the intravenous administration of EP-3533. The image acquisition parameters were as follows: TE = 3.6 ms; TR = 8.6 ms; field of view (FOV) = 52 mm × 70 mm; matrix = 192 × 256; slice thickness = 2.0 mm.

### Image Analysis

To avoid measurement bias, the targeted segments on MR images were marked using location-by-location evaluation by two radiologists who had 12 and 6 years of experience in gastrointestinal MR imaging. These two radiologists were blinded to the histopathological information. They delineated the region of interest (ROI) on the designated segments. The ROIs covered the layers and entire circumference of the bowel walls in axial sections, and the inter- and extra-gut components were avoided. The distribution of CD lesions was characterized as segmental and leaping. Therefore, 1–2 discrete targeted bowel segments (interval >2 cm) were selected according to the extent and severity of the bowel lesions from one single rat, and the analysed segment results were used as independent data for statistical analysis.

The intestinal contrast-to-noise ratio (CNR) was calculated as the difference in the signal-to-noise ratio between the bowel and muscle ROIs. The time-dependent change in CNR (ΔCNR = CNRpost – CNRpre) following contrast agent injection was determined using the dynamic MR images. Quantitative *T_1_* maps were automatically reconstructed on a voxel-by-voxel basis when the *T_1_* value was first evaluated using the MapIt processing tool (MapIt software; Siemens, Erlangen, Germany) based on a dual flip-angle (2° and 12°) 3D gradient echo sequence.

### Histopathological Analysis

Animals were sacrificed immediately after MR examination, and the bowel tissue was prepared for analysis. The anus and gross lesions were chosen as the positioning landmarks to select specimens to evaluate the identity between MR imaging and histopathological analysis. Subsequently, the bowel specimens were fixed with 4% paraformaldehyde, embedded in paraffin, cut into 4 μm slices, and stained with haematoxylin and eosin (HE) and Masson’s trichrome according to standard procedures. The histological classification of fibrosis was analyzed based on a semi-quantitative scoring system as described previously (Li et al., 2018).

### Statistical Analysis

Statistical analyses were performed using SPSS v20.0 (IBM Inc., Armonk, NY, United States), with *p* < 0.05 indicating a significant difference. The data were reported as means ± standard deviation (SD). The normality of data was evaluated using the Shapiro-Wilk test. All the tests were two-sided comparisons. Two-way repeated-measures analysis of variance (ANOVA) was performed to evaluate the effect of the time (before and after injecting the contrast agent) and time-group interaction. Spearman’s rank test was performed to determine the correlation between the fibrosis scores and MR index (ΔCNR and the *T*_*1*_ value). A coefficient r absolute value <0.25 was recognized as a poor correlation, 0.25–0.50 as moderate, 0.50–0.75 as good and larger than 0.75 as excellent. Receiver operating characteristic (ROC) curve analysis was performed, and the area under the curve (AUC) was used to determine the diagnostic accuracy of ΔCNR and the *T*_*1*_ value to evaluate different degrees of bowel fibrosis. AUCs >0.85, 0.7–0.85, and <0.7 indicated high, moderate, and low accuracy, respectively.

## Results

### Relaxivity of EP-3533

**FIGURE 1:**
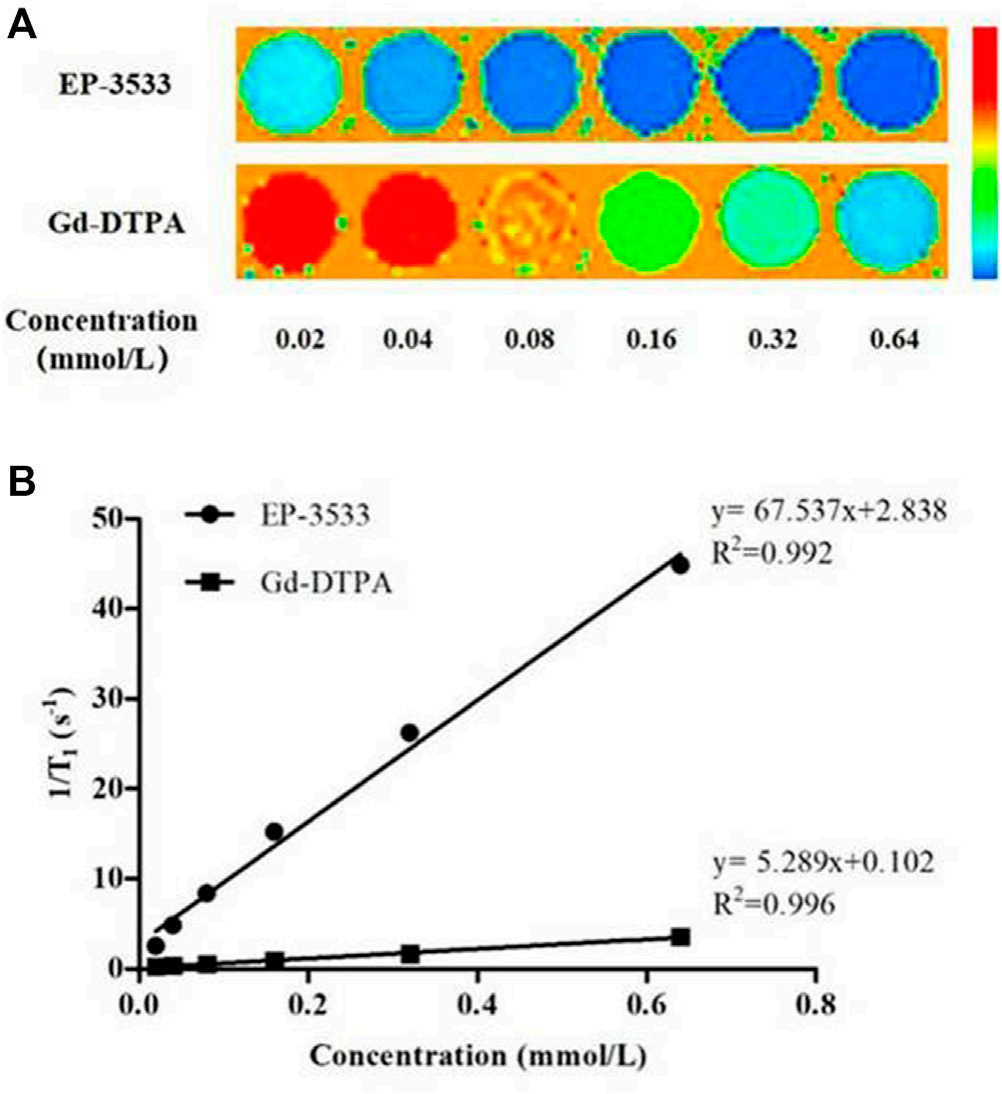
The longitudinal relaxivity of EP-3533 and Gd-DTPA. *T*_*1*_ maps **(A)** of EP-3533 and Gd-DPTA were obtained in aqueous solution at 25°C at 3.0-T field strengths. The slopes of the linear fitting equation **(B)** represents the relaxivities of the contrast agents.

The relaxivity of EP-3533, expressed on a per-molecule basis, was measured in aqueous solution at 25°C under 3.0-T field strengths. The relaxivity of EP-3533 was approximately 12.77 times higher than that of Gd-DTPA per molecule (r_1_ = 5.289 L/mmol·s) with r_1_ = 67.537 L/mmol·s (Figure 1).

### Characterization of Bowel Fibrosis in the TNBS-Induced Rat Model

To characterize the bowel fibrosis in the TNBS-induced rat model, pathological scoring on tissue sections was performed. In total, 33 bowel specimens from 22 rats were acquired for histopathological evaluation. In 13 rats imaged with EP-3533, 18 bowel specimens were obtained that included one specimen with a fibrosis score of 0, 4 specimens with a fibrosis score of 1, 9 specimens with a fibrosis score of 2 and 4 specimens with a fibrosis score of 3. In 9 rats imaged with Gd-DTPA, 15 bowel specimens were obtained that included one with no fibrosis (score 0), 3 with a fibrosis score of 1, 10 with a score of 2 and one with severe fibrosis (score 3). Overall, 2 specimens had a fibrosis score of 0, 7 had a score of 1, 19 had a score of 2, and 5 specimens with severe fibrosis had a score of 3 among 33 bowel specimens.

### Molecular MR Imaging of Fibrotic Bowel Segments

**FIGURE 2:**
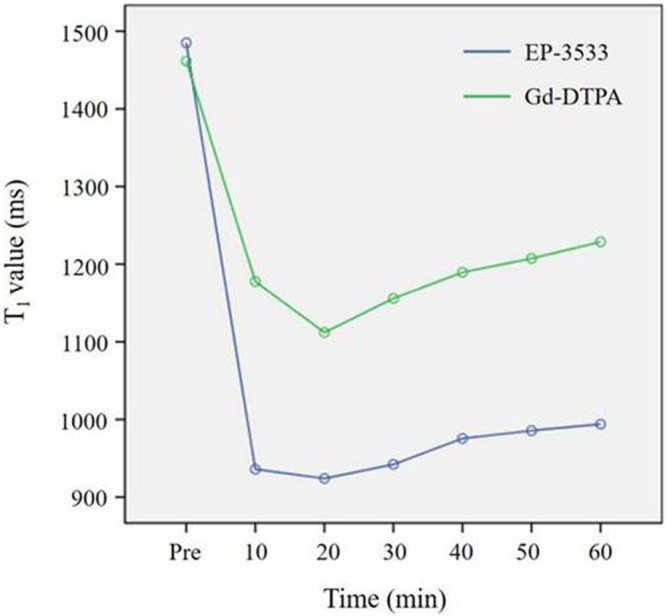
Change in *T_1_* values from the bowel wall according to the time stream after the injection of EP-3533 and Gd-DTPA. To generate longitudinal relaxation rate maps, a series of dual flip-angle (2° and 12°) three-dimensional (3D) gradient echo sequences were also acquired before and 10 min, 20 min, 30 min, 40 min, 50 min and 60 min following the intravenous administration of EP-3533. The image acquisition parameters comprised the following: TE = 3.6 ms; TR = 8.6 ms; field of view (FOV) = 52 mm × 70 mm; matrix =192 × 256; slice thickness = 2.0 mm.

**FIGURE 3:**
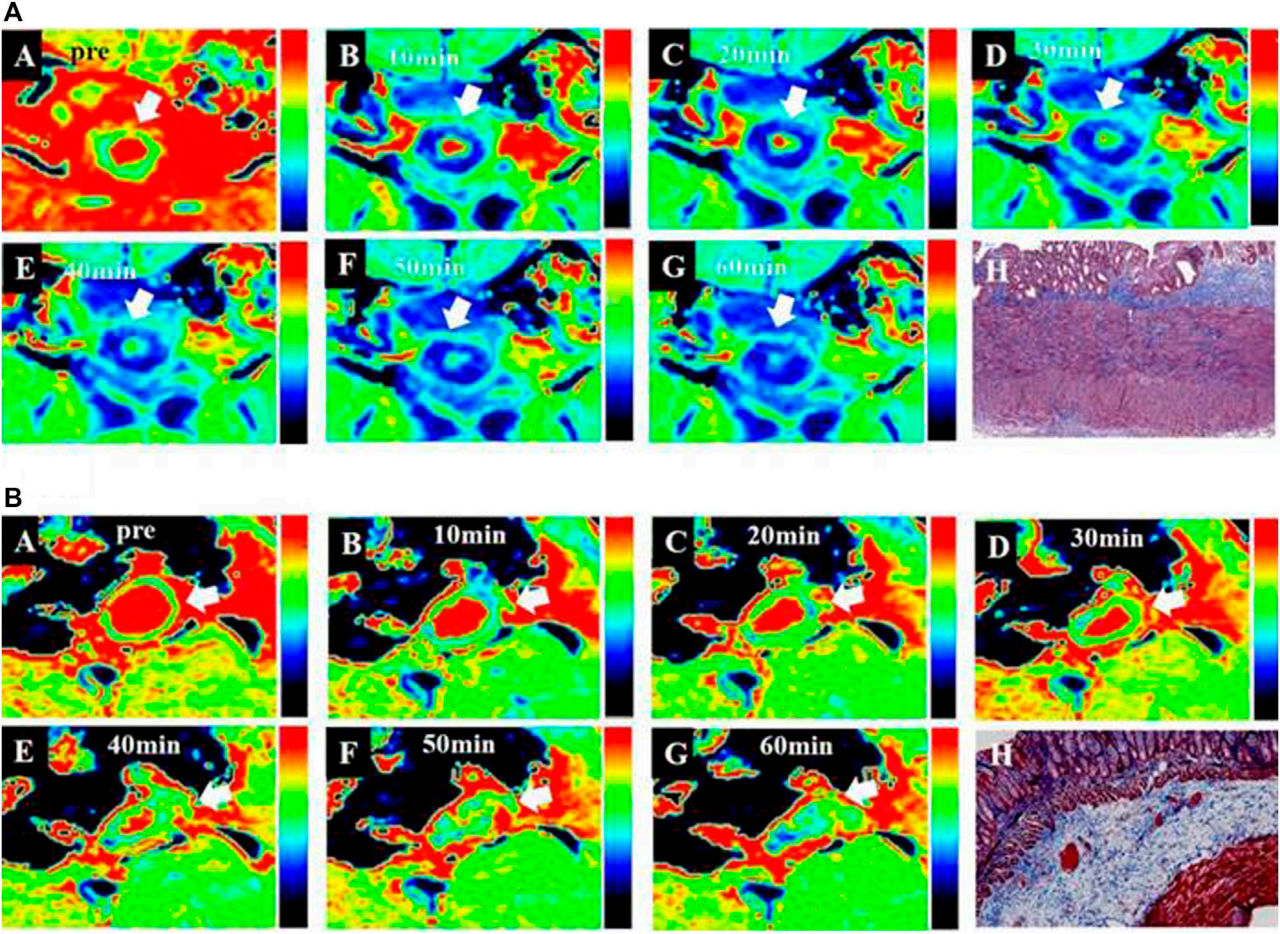
*T*_*1*_ maps of bowel wall fibrosis obtained from MR imaging according to the time stream after contrast media injection. **(A)**, *T*_*1*_ maps from EP-3533 enhanced MR imaging: A to G are the *T*_*1*_ maps of a moderate bowel wall fibrosis before and after injecting EP-3533. Bowel wall fibrosis is depicted in green **(A)** before the injection of EP-3533. After EP-3533 administration, bowel wall fibrosis marked by white arrows showed obvious enhancement in blue in the *T*_*1*_maps **(B–G)**. Masson’s trichrome staining **(H)** depicted moderate fibrosis (score = 2). **(B)**, *T*_*1*_ maps from Gd-DTPA enhanced MR imaging:A to G are the *T*_*1*_ maps of moderate bowel wall fibrosis before and after injecting Gd-DTPA. Bowel wall fibrosis is also depicted in green **(A)** before injecting Gd-DTPA. After Gd-DTPA administration, the bowel wall fibrosis marked by white arrows showed mild enhancement in green in the *T*_*1*_ maps **(B–G)**. Masson’s trichrome staining (P) depicted moderate fibrosis (score = 2).

A dramatic change in the *T*_*1*_ value of the TNBS-induced CD rats was observed every 10 min after contrast agent injection for 1 h. Two-way repeated-measures ANOVA showed that the difference of interactive effect on treatment (different contrast agents) and time was significant (F = 6.422; *p* = 0.001). Our results indicated that the change in the *T*_*1*_ value of the bowel wall was time dependent and varied from EP-3533 to Gd-DTPA. During scanning with the administration of EP-3533, the *T*_*1*_ value decreased sharply at 20 min post EP-3533 injection and then increased slowly. In contrast, the *T*_*1*_ value of Gd-DTPA-enhanced MR imaging decreased slowly at 20 min, and the increase in the *T*_*1*_ value was more obvious than that with EP-3533 imaging (Figures 2, 3). In addition to the result of significant interaction between the imaging time and contrast media, the single effect of time and contrast agent was also analyzed separately. First, a significant difference in the *T*_*1*_ value was obtained between the EP-3533 and Gd-DTPA groups (F = 16.478; *p* < 0.001). Overall, the *T*_*1*_ value of the bowel wall in the EP-3533 group was 184.348 ms (95% CI: 91.601–227.095), which was shorter than that in the Gd-DTPA group. The *T*_*1*_ value variability showed significant difference between the EP-3533 and Gd-DTPA groups on the scan time after injection (all *p* < 0.002). Furthermore, the *T*_*1*_ value variability was closely correlated with the scan time (F = 9.787; *p* < 0.001) in both the EP-3533 (F = 7.468; *p* = 0.004) and Gd-DTPA (F = 4.185; *p* = 0.015) groups (Table 1).

**TABLE 1:** *T*_*1*_ value of the bowel wall before and after molecular probe (EP-3533) and GD-DTPA injection.

		Pre	10 min	20 min	30 min	40 min	50 min	60 min	Total	*F*	*p*
EP-3533	‾x	1,484.328	936.336	924.074	942.444	975.428	985.646	994.084	1,034.612	7.468	0.004
	SD	190.538	164.516	154.606	157.284	168.425	158.062	168.095	30.617		
Gd-DTPA	‾x	1,462.531	1,177.135	1,111.893	1,155.560	1,189.651	1,207.214	1,228.670	1,218.960	4.185	0.015
	SD	190.769	157.514	153.069	137.265	140.672	148.093	172.806	33.539		
Total	‾x	1,474.420	1,045.790	1,009.447	1,039.315	1,072.802	1,086.358	1,100.714	1,126.786	9.787^a^	0.000^a^
	SD	187.963	200.147	178.797	181.654	188.311	188.201	201.294	22.705		
*F*		0.136	18.680	11.873	16.977	14.871	16.471	15.091	16.478^b^	6.442^c^	0.001^c^
*p*		0.714	0.000	0.002	0.000	0.001	0.000	0.001	0.000^b^		

a *F* statistic and *p* value of the main effect of time.

b *F* statistic and *p* value of the main effect of the contrast agent.

c F statistics and *p* values for interaction effects.

**FIGURE 4:**
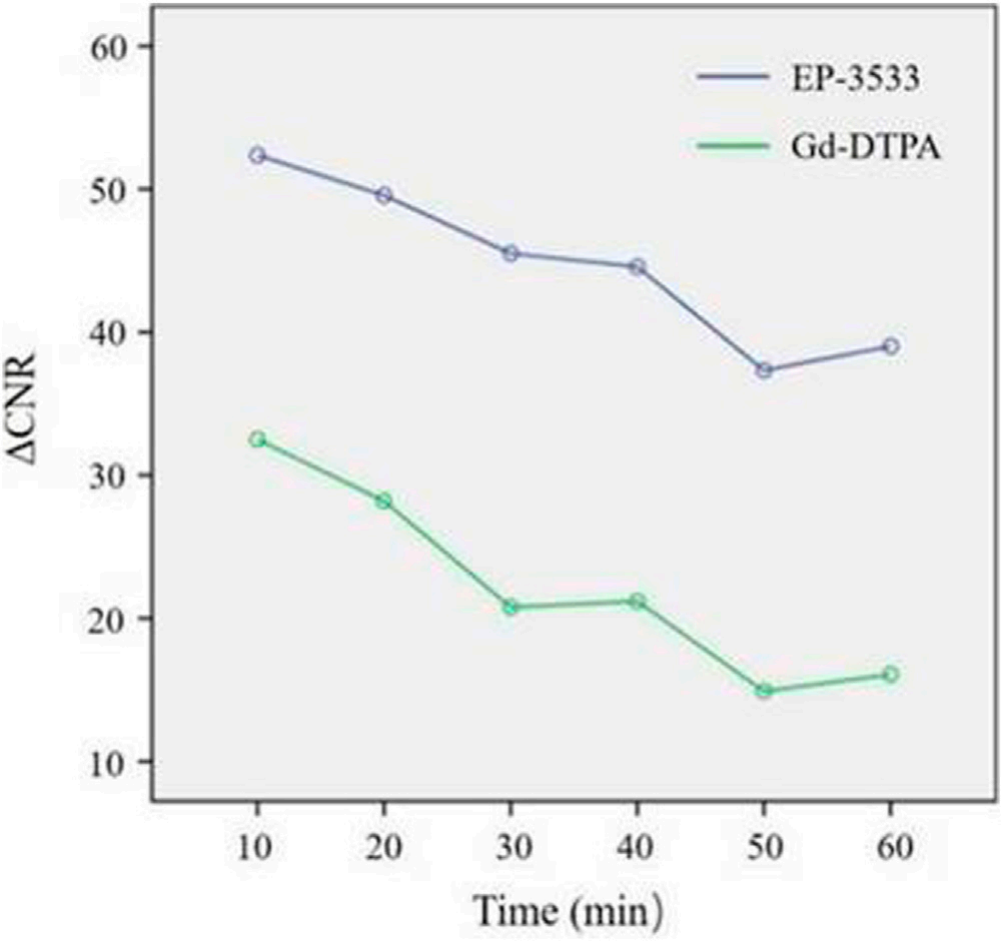
Change inΔCNR of the bowel wall according to the time stream after the administration of EP-3533 and Gd-DTPA. The bowel contrast-to-noise ratio (CNR) was calculated as the difference in the signal-to-noise ratio between the bowel and muscle ROIs. The time dependence of the change in CNR (ΔCNR = CNR_post_–CNR_pre_) following contrast agent injection was determined using dynamic MR images.

**FIGURE 5:**
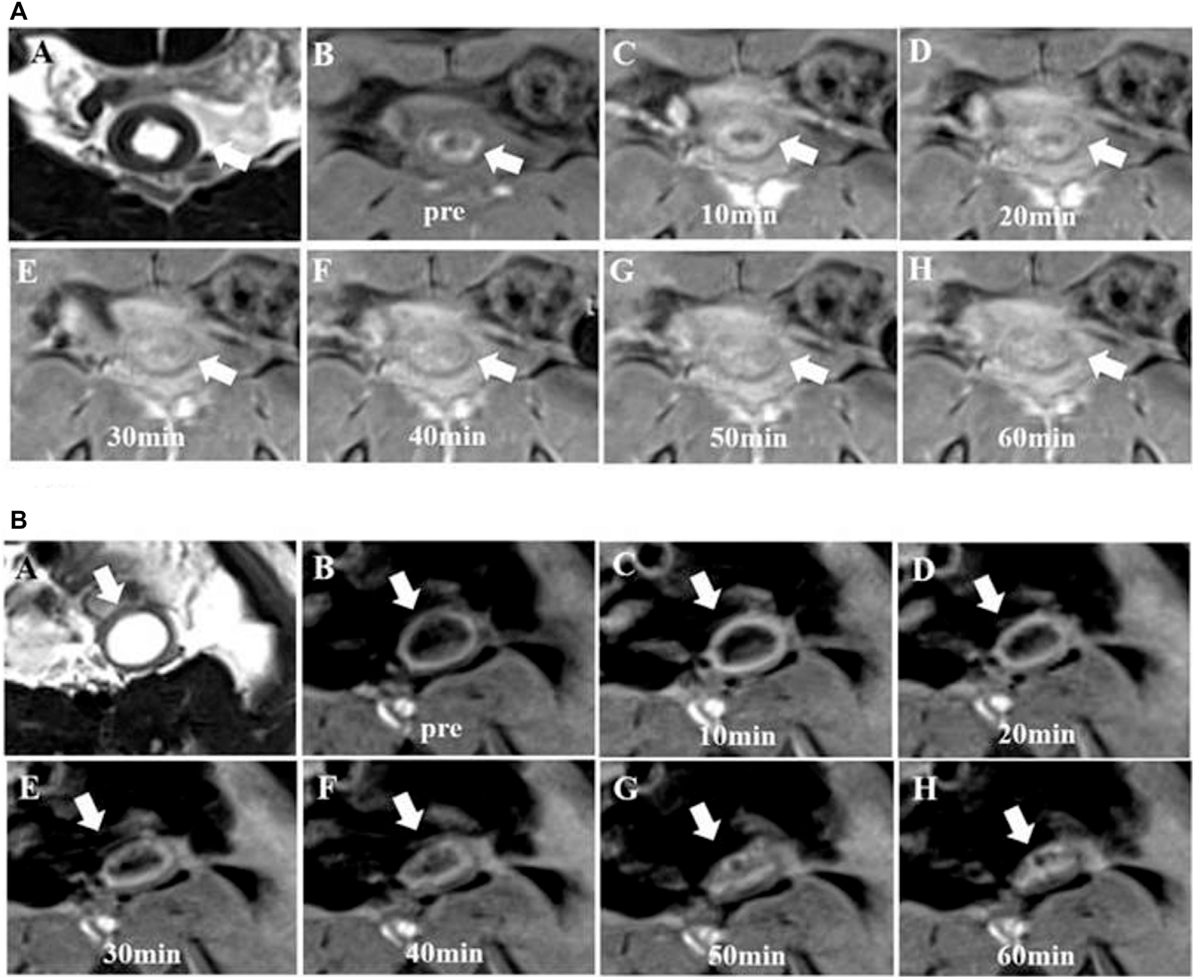
Signal intensity maps of the fibrotic bowel wall obtained from MR imaging. A to H are maps of moderate bowel wall fibrosis. **(A)**, T2-weighted imaging enhanced from EP-3533 enhanced MR imaging: T2-weighted imaging **(A)** and T1-weighted imaging **(B)** showed thickening of the bowel wall. The mucosa of the bowel wall showed hyperintensity on T1-weighted imaging due to the mucus adhering to the bowel wall surface. After EP-3533 injection, the bowel wall fibrosis marked by the red arrows showed obvious enhancement **(C–G)**. **(B)**, T2-weight imaging from Gd-DTPA enhanced MR imaging: A to G are the maps of the other moderate bowel wall fibrosis pre- and post-injection of Gd-DTPA. After Gd-DTPA administration, the bowel wall fibrosis showed slight enhancement.

Regarding the analysis of ΔCNR, no significant interactive effect was found between the contrast agent and time effects (F = 0.261; *p* = 0.868). Although ΔCNR depended on the scan time, the variability according to the scan time showed the same trend between the EP-3533 and Gd-DTPA groups (Figures 4, 5). Therefore, the effects of contrast and scan time were analyzed in our study. ΔCNR depended both on the contrast agent (F = 15.281; *p* < 0.001) and scan time (F = 3.656; *p* = 0.013). However, ΔCNR in the EP-3533 group was more apparent; it decreased with the injection time, with the highest ΔCNR obtained 10 min post injection (Table 2).

**TABLE 2:** ΔCNR of the bowel wall before and after contrast agent injection.

		10 min	20 min	30 min	40 min	50 min	60 min	*F*	*p*
EP-3533	‾x	52.312	49.511	45.473	44.545	37.279	38.969		
	SD	22.467	19.255	21.119	17.821	22.923	23.328		
Gd-DTPA	‾x	32.577	28.271	20.824	21.223	14.955	16.701		
	SD	14.952	17.485	15.485	13.308	12.291	16.701		
Total	‾x	43.342	39.856	34.269	33.945	27.132	28.591	3.656^a^	0.013^a^
	SD	21.576	21.120	22.298	19.628	21.741	23.332		
*F*	15.281^b^							0.261^c^	0.868^c^
*p*	0.000^b^								

a *F* statistic and *p* value of the main effect of time.

b *F* statistic and *p* value of the main effect of contrast agent.

c F statistics and *p* values for interaction effects.

### Efficacy of Molecular MR Imaging to Diagnose Bowel Fibrosis

**FIGURE 6:**
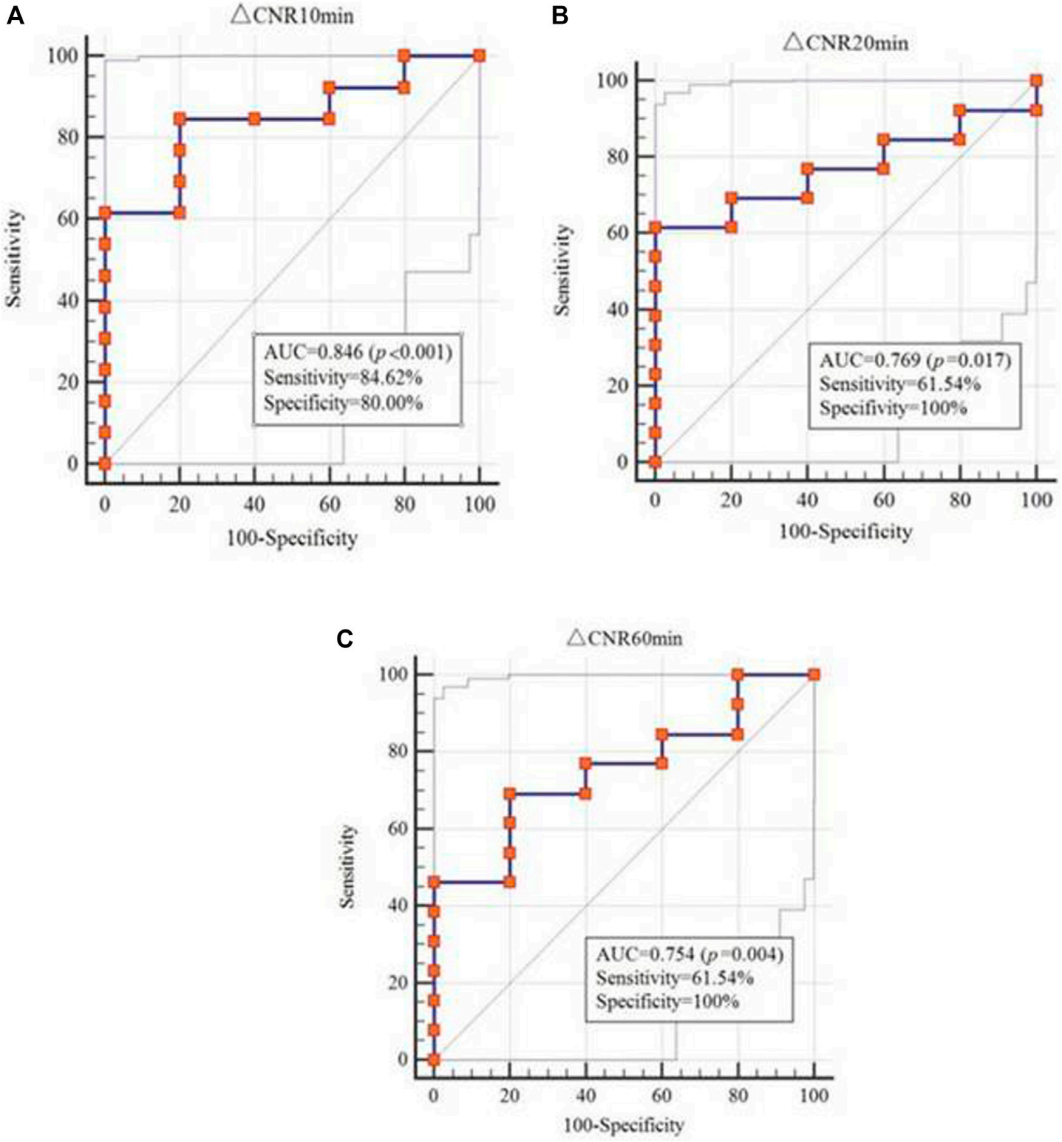
ROC analysis of ΔCNR to differentiate between none-to-mild and moderate-to-severe fibrosis.

To determine the diagnostic effect of MR imaging using different enhanced media and scan times, the *T*_*1*_value and ΔCNR were analyzed. The *T_1_* value obtained from EP-3533 MR imaging (AUC = 0.785; 95% CI: 0.531–0.940) showed a better correlation with fibrosis than that from Gd-DTPA (AUC = 0.545; 95% CI: 0.276–0.797), which was measured 10 min after enhanced media administration (Table 3). ΔCNR obtained from EP-3533 MR imaging showed a significant correlation with the degree of fibrosis (*r* = 0.538; *p* = 0.021) at the time point of 10 min post injection. The AUC of ΔCNR at 10 min post EP-3533 injection was 0.846 (95% CI: 0.601–0.970; *p* = 0.0003; sensitivity = 84.62%; specificity = 80%). Additionally, ΔCNR at 20 and 60 min showed a meaningful correlation with fibrosis, despite low sensitivity (61.5% and 69.2%) (Figure 6). By contrast, ΔCNR of Gd-GDPA-enhanced MR imaging showed no correlation with the degree of fibrosis (*p* > 0.05).

**TABLE 3:** Diagnostic performance of the *T*_*1*_ value and ΔCNR in differentiating bowel fibrosis at each time point after contrast agent injection.

		EP-3533					Gd-DTPA					
		AUC (95% CI)	*z*	*P*	Sensitivity	Specificity	AUC (95% CI)	*z*	*p*	Sensitivity	Specificity
*T*_*1*_value	10 min	0.785 (0.531, 0.940)	1.697	0.0897	100.00	60.00	0.545 (0.276, 0.797)	0.239	0.8112	54.55	75.00	
	20 min	0.646 (0.390, 0.852)	0.751	0.4529	84.62	60.00	0.705 (0.420, 0.905)	1.312	0.1896	72.73	75.00	
	30 min	0.677 (0.420, 0.874)	0.989	0.3226	84.62	60.00	0.523 (0.257, 0.779)	0.113	0.9103	81.23	50.00	
	40 min	0.538 (0.293, 0.771)	0.210	0.8339	69.23	60.00	0.705 (0.420, 0.905)	1.203	0.2291	45.45	100.00	
	50 min	0.523 (0.280, 0.759)	0.112	0.9106	92.31	40.00	0.591 (0.315, 0.830)	0.373	0.7094	100.00	50.00	
	60 min	0.508 (0.267, 0.746)	0.036	0.9710	100.00	40.00	0.500 (0.239, 0.761)	0.000	1.0000	100.00	25.00	
ΔCNR	10 min	0.846 (0.601, 0.970)	3.580	0.0003	84.62	80.00	0.523 (0.257, 0.779)	0.104	0.9174	18.18	50.00	
	20 min	0.769 (0.514, 0.931)	2.396	0.0166	61.54	100.00	0.636 (0.356, 0.862)	0.598	0.5500	72.73	75.00	
	30 min	0.738 (0.482, 0.913)	1.814	0.0697	46.15	100.00	0.545 (0.276, 0.797)	0.201	0.8407	81.82	50.00	
	40 min	0.685 (0.427, 0.879)	1.320	0.1869	38.46	100.00	0.500 (0.239, 0.761)	0.000	1.0000	81.82	50.00	
	50 min	0.662 (0.405, 0.863)	1.126	0.2603	38.46	100.00	0.523 (0.257, 0.779)	0.111	0.9120	100.00	25.00	
	60 min	0.754 (0.498, 0.922)	2.060	0.0394	69.23	80.00	0.636 (0.356, 0.862)	0.598	0.5500	72.73	75.00	

## Discussion

Clinically, CD-associated structural change as symptomatic stricture is frequently treated with anti-inflammatory therapy regardless of whether the stricture is caused by inflammation or fibrosis. Because of the lack of specific treatment options, CD overtreatment is commonly not a concern (Bettenworth et al., 2019). The diagnosis of CD-associated strictures on cross-sectional imaging, such as contrast-enhanced CT (CE-CT), depends on the repeatability to reconstruct high-quality multiplanar images that illustrate the entire large and small bowel in patients independent of body size. However, CE-CT is not a superior technique to distinguish the degree of stricture of inflammation and fibrosis (Chiorean et al., 2007; Adler et al., 2012; Bettenworth et al., 2019). In comparison, MR enterography (MRE) based on MR techniques has excellent capability to assess the degree of inflammation. However, the differentiation of fibrosis from inflammation in CD strictures through currently available cross-sectional imaging techniques remains challenging (Rimola et al., 2011; Rimola et al., 2015; Pous-Serrano et al., 2017; Li et al., 2018; Wagner et al., 2018; Wilkens et al., 2018).

Fibrosis is characterized by excessive accumulation of ColI. Therefore, a type Ⅰ collagen-targeted MRI probe (EP-3533) was employed to evaluate CD-associated bowel fibrosis in our study, based on the fact that EP-3533 offered good diagnostic efficiency in liver fibrosis (Caravan et al., 2013; Farrar et al., 2015) and pulmonary fibrosis (Caravan et al., 2013; Ding et al., 2014).

Because the MR signal intensity is affected by many technical factors, the relationship between the MR signal intensity and gadolinium concentration is not linear (Katsube et al., 2011; Ding et al., 2014). *T*_*1*_ mapping is a robust MRI technique that allows measurement of the longitudinal relaxation time (*T*_*1*_ value) that reflects the inherent characteristics of tissue and depicts even small changes within tissues (Taylor et al., 2016). A previous study demonstrated that measurement of the *T*_*1*_ relaxation time using *T*_*1*_ mapping is more reliable and objective, allowing the quantitative evaluation of contrast agent uptake by liver parenchyma (Feier et al., 2013). Additionally, ΔCNR is calculated based on the MR signal intensity, and it is a common imaging indicator for contrast agent enhancement. A previous study showed that EP-3533 is the most sensitive contrast media for evaluating the enhancement effect of EP-3533 compared with other one (Caravan et al., 2013). Therefore, the *T*_*1*_ value and ΔCNR were calculated in our study to analyze the diagnostic efficiency of enhanced MR imaging in CD-associated fibrosis regarding variations in enhanced media and scan time.

First, our results have indicated that EP-3533 supplied a better relaxivity (r1 = 67.537 L/mmol·s) than Gd-DTPA (r1 = 5.289 L/mmol·s), supporting the hypothesis that EP-3533 is more suitable for imaging CD-associated fibrosis than Gd-DTPA.

Furthermore, our study demonstrated that the best scanning time to analyze fibrosis was 10 min post EP-3533 injection because the *T*_*1*_ value offered a correlation with the histopathological results to analyze CD-associated fibrosis (AUC = 0.785; 95% CI: 0.531–0.940). By contrast, the AUC of Gd-DTPA was only 0.545 (95% CI: 0.276–0.797). Our results of two-way repeated-measures ANOVA also indicated that the change in the *T*_*1*_ value of the bowel wall was dependent on time and varied from EP-3533 to Gd-DTPA (F = 6.422; *p* = 0.001). A significant difference in the *T*_*1*_ value was obtained between the EP-3533 and Gd-DTPA imaging groups, with *p* < 0.001 (F = 16.478). The variation in the *T*_*1*_ value according to the scan time between EP-3533 and Gd-DTPA was also significant (all *p* < 0.002).

Additionally, ΔCNR obtained from EP-3533 MR imaging exhibited the most significant correlation with the degree of fibrosis determined from histopathological results with an r value of 0.532 and an AUC of 0.846 (95% CI: 0.601–0.970; *p* = 0.0003), indicating a diagnostic effect comparable to that of duplex US, as shown by Ripollés et al. (2013) and better than that of Gd-DTPA (AUC = 0.523; 95% CI: 0.257–0.779). Additionally, when assessing bowel fibrosis in CD, a sensitivity of 84.62% and a specificity of 80% were achieved. The fibrosis-targeted MR imaging probe supplied a better sensitivity than MRE with a sensitivity of 61% or PET-MRE with a sensitivity of 66.7% (Pellino et al., 2016; Wagner et al., 2018).

A therapeutic approach against fibrosis must be applied for prolonged periods of time with minimal side effects. Therefore, inhibition of ColI production only in fibrotic lesions and constitutive ColI synthesis is the optimal antifibrotic therapy strategy. Additionally, ColI is one of the most stable proteins in the human body, with a half-life of 4–12 months. Its fractional synthesis rate (defined as % synthesis per day) is very low in healthy organs, in contrast to synthesis in fibrosis, where ColI production increased several hundred-fold (Stefanovic et al., 1997; Lindquist et al., 2000). ColI is a suitable target for the diagnosis and treatment of tissue fibrosis, including in patients with CD. Our results demonstrated that the ColI-targeted MR imaging probe EP-3533 is significantly correlated with the histopathological outcome of fibrosis, suggesting that EP-3533 can monitor the therapeutic response of tissue fibrosis under ColI-targeted treatment.

In conclusion, our study reveals that the ColI-targeted imaging probe EP-3533 is more effective than Gd-DTPA in evaluating the progression of fibrosis in bowel fibrosis. Our results suggest that EP-3533 could be used to monitor the ColI-targeted therapeutic response of tissue fibrosis.

## Data Availability

The original contributions presented in the study are included in the article/Supplementary Material, further inquiries can be directed to the corresponding authors.

## Abbreviations

AUC, area under the curve; CD, Crohn’s disease; CNR, contrast-to-noise ratio; ColІ, type І collagen; CT, computed tomography; ECM, extracellular matrix; FOV, field of view; Gd-DTPA, gadopentetatedimeglumine; HE, haematoxylin and eosin; MRI, magnetic resonance imaging; ROI, region of interest; SD, standard deviation; TE, echo time; TNBS, 2, 4,6-trinitrobenzene sulfonic acid; TR, repetition time; US, ultrasound.
